# Rapid Detection of *Chlamydia trachomatis *and Typing of the Lymphogranuloma venereum associated L-Serovars by TaqMan PCR

**DOI:** 10.1186/1471-2334-8-56

**Published:** 2008-04-30

**Authors:** Anke Schaeffer, Birgit Henrich

**Affiliations:** 1 Institute of Medical Microbiology and Hospital Hygiene, Clinical Center of Heinrich-Heine University, Moorenstrasse 5, 40225 Duesseldorf, Germany

## Abstract

**Background:**

Infection due to *Chlamydia trachomatis *is the most common sexually transmitted bacterial disease of global health significance, and especially the L-serovars causing lymphogranuloma venereum are increasingly being found in Europe in men who have sex with men.

**Results:**

The design and evaluation of a rapid, multiplex, real-time PCR targeting the major outer membrane protein (*omp-1*) -gene and a L-serovar-specific region of the polymorphic protein H (*pmp-H*) -gene for the detection of *Chlamydia trachomatis *is reported here. The PCR takes place as a single reaction with an internal control. For L1-, L2- and L3-serovar differentiation a second set of real-time PCRs was evaluated based on the amplification of serovar-specific *omp-1*-regions. The detection limit of each real-time PCR, multiplexed or not, was 50 genome copies per reaction with an efficiency ranging from 90,5–95,2%.

In a retrospective analysis of 50 ocular, rectal and urogenital specimens formerly tested to be positive for *C. trachomatis *we identified six L2-serovars in rectal specimens of HIV-positive men, one in a double-infection with L3, and one L2 in a urethral specimen of an HIV-negative male.

**Conclusion:**

This unique real-time PCR is specific and convenient for the rapid routine-diagnostic detection of lymphogranuloma venereum-associated L-serovars and enables the subsequent differentiation of L1, L2 and L3 for epidemiologic studies.

## Background

*Chlamydia trachomatis *is a sexually transmitted, obligate intracellular human pathogen leading to severe ocular and urogenital tract infections. At present this heterogeneous species is divided into more than 20 serovars [1,2]. The serovars A, B, Ba and C cause trachoma, a chronic ocular disease found predominantly in developing countries, whereas the serovars D, Da, E, F-I, Ia, J, Ja and K are the aetiological agents of oculo-genital infections. While the serovars A-K are usually non-invasive; the LGV-serovars L1, L2, L2a, L2b and L3 are responsible for the sexually transmitted disease (STD) lymphogranuloma venereum, a severe invasive disease with preference for lymph tissue. Infection can result in suppurative proctitis and lymphadenitis [3,4]. Genetic analysis has revealed a further class of subtypes, the so-called mosaic-types. There are at present five different mosaic types, one classified as B/D- [5], three as L1/L2- [6] and a single Ba/D-mosaic [7]. These mosaics have been proposed to emanate from recombination events of the respective serovars.

For many years typing and diagnosis of *C. trachomatis *has been based on the immunological detection of the major outer membrane protein (MOMP) with subsequent expansion into genetic analyses, particularly the detection of the MOMP encoding gene, *omp-1*. The conserved regions of the *omp-1 *gene were used for the species-specific detection of *C. trachomatis *and four variable regions, VR1 to VR4 for sero- and genotyping [8-13].

To improve the sensitivity of chlamydial detection multi-copy templates of 16S rDNA or a species-specific cryptic plasmid of *C. trachomatis *with 10 copies per cell have been targeted [14]. Real-time PCR assays have been developed targeting *omp-1 *[15], the cryptic plasmid [16,17] or the *pmp-H *gene [18,19].

In the molecular diagnostic of lymphogranuloma venereum targeting the *pmp-H *gene has predominated as all the LGV-serovars possess a unique gap of 36 nt which is absent in the other serovars. Furthermore a *pmp-H*-based restriction fragment length polymorphism (RFLP) pattern clusters the organism into distinct groups closely associated with clinical disease: ocular, urogenital and LGV [20]. Chen and co-worker published in 2006 an evaluation of a real-time, multiplex PCR targeting the *pmp-H *gene resulting in the simultaneous detection of LGV and differentiation from the other serovars [18].

Ongoing studies have revealed a progressive dissemination of the L2/L2b serotype in Europe among men who have sex with men (MSM) [21-23]. Since 2003, 78 males with LGV have been reported in Germany, 61 were confirmed as genotype/serovar L2 [24]. For the rapid detection of L2/L2b, a number of real-time PCR assays have recently been developed [25-27], but so far no one-step real-time PCR enabling the simultaneous differentiation of L1 and L3 has been published.

After the publication of a case report describing the diagnosis of L1 in a MSM with proctitis in 1995 [28], we started to develop a PCR assay allowing the detection and differentation of the LGV-serovars. In this study we present the development and evaluation of a multiplex real-time PCR targeting the *omp-1 *gene for species-specific detection of *C. trachomatis *and the *pmp-H *gene for LGV typing. In addition, three real-time PCR assays were developed for the subsequent LGV differentiation into the L1-, L2- and L3-serotypes.

## Methods

### Bacterial strains

To investigate the analytical specificities of the primers and probes sixteen isolates were used representing the serovars and serovariants of *Chlamydia trachomatis *(Table 3), and additionally *C. muridarum*, *C. pneumoniae*, *C. pecorum*, *C. psittaci *and twenty other microorganisms that normally reside in the human oropharynx, the urogenital and perianal tract. These microorganisms corresponded to the gram-positive and gram-negative bacteria and yeast previously described by Morré and coworker [19]: *Acinetobacter baumannii, Campylobacter jejuni, Candida albicans*, *Enterococcus faecalis, Escherichia coli, Streptococcus agalactiae, Haemophilus influenzae*, *Klebsiella pneumoniae, Lactobacillus casei, Mycoplasma genitalium*, *M. hominis, M. pneumoniae, Neisseria meningitidis*, *Pasteurella multocida, Pseudomonas aeruginosa, Salmonella enteritidis, Shigella sonnei, Staphylococcus aureus*, *Ureaplasma parvum *and *U. urealyticum*. Chlamydial DNA was kindly provided by Servaas Morré and DNA extracts of the other microorganisms derived from our diagnostic laboratory, each covering not less than 10 ng DNA/2.5 μl.

### Clinical specimens

The specimens with a request for detection of *C. trachomatis *originated from different departments of the University Clinic of Duesseldorf. Sample collection for routine diagnostic procedures was conducted in adherence with the guidelines of good clinical practice under approval of the Institutional Review Board of the University Clinic Duesseldorf. Written informed content was obtained from all patients. Less than 10% were ocular samples. 43% of the samples emanated from women with clinical symptoms and 7% from women for routine screening in pregnancy. 32% of the specimens derived from departments specialising in sexual transmitted diseases especially of men (andrology (16%); dermatology (6%) and infectious diseases, which also includes the treatment of HIV-infected men, (10%)).

The ocular, urethral, cervical and rectal swabs were transported to the Medical Microbiology in 3 ml M4-transport medium (Remel, UK) which contains 3–4 glass beads. Upon arrival, the specimens were resuspended in the medium by vortexing, and a portion of 1 ml centrifuged at 15,000 × g for 15 min. The supernatant was removed and the sediment washed in 500 μl phosphate-buffered saline, pH 7.3. After further centrifugation the sediment was resuspended in 25 μl 10 mM Tris-(hydroxymethylaminomethan) (Tris)/HCl, pH 7,5. Thereafter 50 μl Proteinase K-solution (100 μg/ml Proteinase K - 0,5% Tween 20 in 10 mM Tris/HCl, pH 7.5) was added and the samples were incubated for 60 min at 56°C followed by inactivation of the Proteinase K at 95°C for 30 min. After a short centrifugation, the samples were ready to be used in PCR. If not tested immediately, they were stored at -20°C.

### Design of Primers and Probes

**Figure 1:**
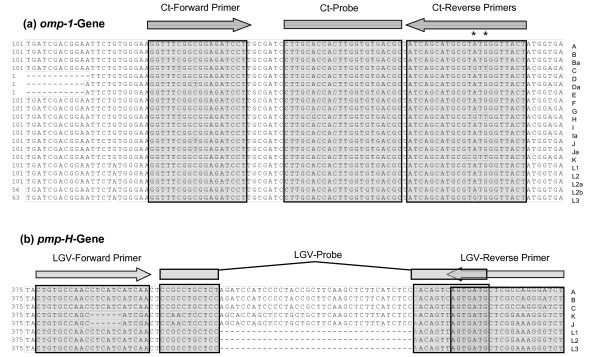
**Alignments of the *omp*-1- (a) and *pmp*-H-gene regions (b) by the use of different *C. trachomatis *serovars A to L.** The corresponding accession numbers are given in Figure 3 (*omp*-1) and in Material and Methods (*pmp*-H). Identical nucleotides are boxed, the binding sites of primers and probes are marked above. *, difference in the Ct-R1 and -R2 primers.

Multiple-sequence alignments were performed with the MegAlign program of the sequence analysis software of DNASTAR (see Figures 1 and 3). The Primer Express Software of Applied Biosystems (Foster City, CA, USA) was used for the final design of specific primers and probes. The *omp*-1-sequences (Figure 3) and *pmp*-H sequences (serovars A (AY184155), B (AY184156), C (AY184158), J (AY184165), K (184166), L1/2/3 (AY184167/8/9)) were retrieved from the database at the National Center for Biotechnology Information (see Availability and requirements section for URL). Primers and probes derived from MWG-Biotech (Ebersberg, Germany), metabion (Planegg-Martinsried, Germany) or Eurogentec (Seraing, Belgium).

### PCR assays

The CT/LGV-real-time PCR assay was carried out in a total volume of 25 μl consisting of 1 × Eurogentec MasterMix without ROX, 5 mM MgCl_2_, Amperase (Eurogentec, Seraing), 150 nM CT-F primer, 115 nM each CT-R (-R1 and -R2) primer and 100 nM FAM-labelled CT-probe, 150 nM of the LGV primers LGV-F and -R and 100 nM of the HEX-labelled LGV-probe and 2.5 μl of template DNA. As internal inhibition control 10^3 ^copies of IK_C plasmid and 100 nM TexasRed-labelled Dros-probe (for the detection of internal control plasmid amplification) were added.

The L-serovar differentiation was either performed in four monoplex or in two duplex real-time PCR assays (as indicated in the Results section). The L1/L2- and the L3/GAPDH-real-time PCR were each carried out in a total volume of 25 μl consisting of 1 × Eurogentec MasterMix without ROX, 5 mM MgCl_2_, Amperase, 150 nM forward and reverse primers, 100 nM labelled probes (see Table 1 for the different fluorophores) and 2.5 μl of template DNA. The conditions of the monoplex PCR assays were the same other than a twofold concentration of primers and probes.

**Table 1:** Primers and probes used in this study

PCR	Gene	SV	Acc-No.	Region/nt	Primer/Probe (5' - 3')
					CT-F: GGT TTC GGC GGA GAT CCT
CT	omp1	all (I)	(AF063200)	124–193	CT-R1: AGT AAC CAA CAC GCA TGC TGA T
					CT-R2: AGT AAC CCA TAC GCA TGC TGA T
					CT-P: FAM-CTTGCACCACTTGGTGTGACGC-TAMRA
IK_CT	ninja	-	AB110070	2079–2051	Dros-P: TexRed-ATG CCT CTT CAC ATT GCT CCA CCT TTC CT-BHQ1
LGV	pmpH	L1	AY184167	377–438	LGV-F: CTG TGC CAA CCT CAT CAT CAA
		L2	AY184168		LGV-R: AGA CCC TTT CCG AGC ATC ACT
		L3	AY184169		LGV-P: HEX-CCG CCT GCT CCA ACA GTT AGT GAT G- BHQ1
K+	GAPDH	-	BT006893	152–221	Gap-F: CCA CCC ATG GCA AAT TCC
					Gap-R: ATG GGA TTT CCA TTG ATG ACA AG
					Gap-P: FAM- TGG CAC CGT CAA GGC TGA GAA CG- BHQ1
L1	omp1	L1	DQ064294	449–532	L1-F: CAG CAT CTT TCA ACT TAG TTG GGT TA
					L1-R: AGC TCA TAT TTG GTA CAG CAT CCT T
					L1-P: HEX-TCG GAG ATA ATG AAA ATC AAA GCA CGG TCA-BHQ1
L2		L2	DQ064295	449–542	L2-F: CAG CAT CTT TCA ACT TAG TTG GGT TAT
					L2-R: TGA TCT AAG CTC ATA TTT GGT ACA AGC TTA
					L2-P: FAM-CGG AGA TAA TGA GAA CCA TGC TAC AGT TTC AGA-BHQ1
L3		L3	DQ064296	456–544	L3-F: CGC TTC CTT CAA CTT AGT TGG ATT
					L3-R: TCA AAG CAG TGT TAG GAA CAA GCT
					L3-P: TET-TTC GGA ACA AAA ACA CAA TCT ACT AAC TTT AAT ACA GCG-BHQ1
L123	omp1	L2	DQ064295	388–542	L123-F : TTG GGA TCG TTT TGA TGT ATT CTG TA
					L2-R: TGA TCT AAG CTC ATA TTT GGT ACA AGC TTA
IK-C	ninja	-	AB110070	2035–2080	IK-C-F: *AGT AAC CAA CAC GCA TGC TG*ATT GCA GCT TCG CCA CAG GA^a^
					IK-C-R: *AGT AAC CCA TAC GCA TGC TGA T *GATGCCTCTTCACATTGCTCC^a^

^a ^5'-fused CT-R1/R2 primer region

Thermal cycling conditions for all real-time PCR assays were as follows: 1 cycle at 50°C for 10 min, 1 cycle at 95°C for 10 min followed by 45 cycles at 95°C for 15 sec and 60°C for 1 min. Cycling, fluorescent data collection and analysis were carried out with an iCycler thermocycler from BioRad according to the manufacturer's instructions.

The internal control plasmid IK_C was constructed essentially as described previously by Rosenstraus *et al*. [29]. The IK_C-F and -R primers (Table 1) used in the PCR were composed of the respective 5'-sequence of the CT primers R1 and R2 fused to 3'-sequences of the ninja transposon of *Drosophila simulans *(not found in *Chlamydia trachomatis*). The plasmid pSARM [30] was used as template which consists of a 276 bp sequence of the ninja transposon and contains the 88 bp target sequence. This target sequence is made up of a 44 bp region of the transposon, containing the 29 bp region recognised by the Dros-probe, and flanked by the primer sequences CT-R1 and CT-R2.

IK_C PCR was conducted in 100 μl 1 × Biorad iQ-Supermix with 3 mM MgCl_2_, 1 μM primer pair IK_C-F and -R and 10 ng pSARM plasmid DNA under the following thermal cycling conditions: 1 cycle at 95°C for 5 min followed by 40 cycles at 95°C for 15 sec, 48°C for 30 sec and 72°C for 30 sec.

Conventional L2-PCR was conducted in 100 μl 1 × Biorad iQ-Supermix with 3 mM MgCl_2_, 1 μM primer pair L123-F and L2-R under the same cycling conditions of the real-time PCR.

IK_C amplicon and the L2-PCR amplicon were isolated using the PCR purification kit of Macherey and Nagel and 10–20 ng amplicons were ligated into 25 ng pGemT-easy using the rapid DNA ligation kit from Roche following the manufacturer's instructions and subsequently propagated in *E. coli *DH5α . After cultivation on LB-Amp agar plates, insert-positive clones were identified by restriction analysis and the sequence determined by sequencing using an ABI sequencer using the method of Sanger *et al*. [31].

## Results

### Two genes for a species- and LGV-specific C. trachomatis detection

Over a period of several years we evaluated the usefulness of targeting a species-specific region of the *omp-1 *gene for *C. trachomatis *detection by comparing real-time PCR data with those produced by a commercial immuno-fluorescence test (IFT) from BioMerieux for perianal, urogenital and ocular swabs [32]. The specificity of the TaqMan primers and probe was verified by alignment of the amplified *omp-1 *gene regions of all known 20 serovars. To compensate for the two variant nucleotides within the reverse primer binding site (marked by stars in Figure 1(a)) we used a mixture of reverse primers, CT-R1 and -R2 (see Table 1). In 2005, 26 of 985 specimens were positive in the CT-real-time PCR whereas only 24 were IFT-positive. Both methods showed 91.7 % concordance (data not shown).

To enable a simultaneous detection of LGV-serovars which, in Europe, have increasingly lead to infections in men who have sex with men, we supplemented the CT reaction with LGV detection. As the polymorphic membrane protein H gene, *pmp-H *has a unique deletion in all LGV-serovars we chose this gene as a target for LGV detection. The LGV-primers used corresponded to those designed by Morré and co-workers [19] whereas the deletion-spanning MGB-probe was replaced by a HEX-labelled standard TaqMan probe (Figure 1).

**Figure 2:**
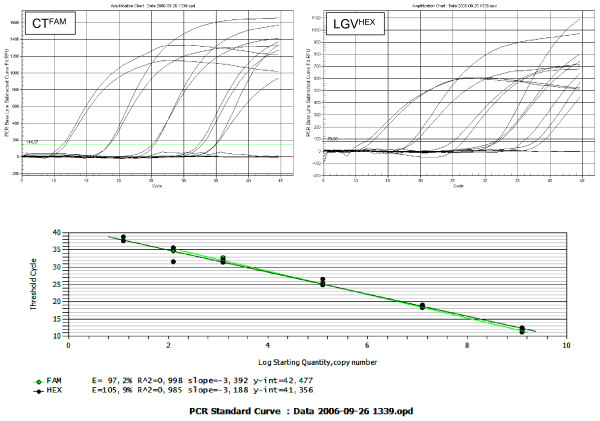
**Amplification charts and standard curves for the CT/LGV multiplexed real-time PCR.** The linear range of the assay was determined using duplicates of 1.25 × [10^9^, 10^7^, 10^5^, 10^3^, 10^2^, 10^1^] copies of each cloned amplicon. The threshold values (C_t_) were plotted against the corresponding copy numbers, and the efficiency, slope and linear regression correlation (r^2^) were calculated for each reaction by the Biorad IQ5 software.

Combined in a duplex real-time PCR assay we first determined the sensitivity and the lower limits of the CT- and LGV-detection using the pGemT-cloned amplicons as template DNA in serial dilutions. All values were measured in duplicate and reproduced in a second run. As shown in Figure 2, the quality of the CT- and LGV-PCR assays was similar in terms of efficiency (93,7% and 90,5%), r^2 ^values (0,988 and 0,993), linear detection range between 10^9 ^and 10^2 ^copies per reaction and detection limits of about 50 genome copies per reaction.

**Figure 3:**
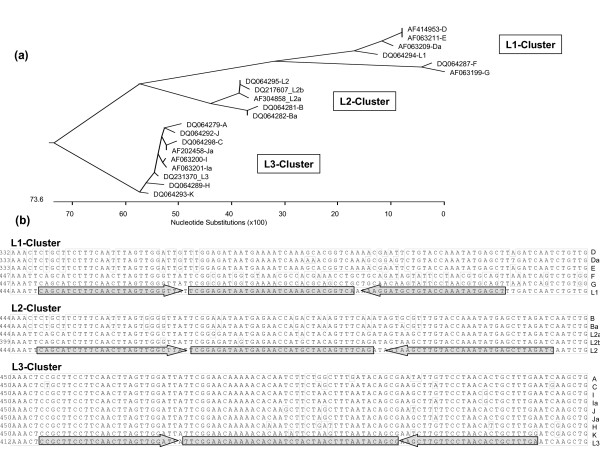
**(a) Phylogenetic tree of the *omp*-1-gene region of 20 *C. trachomatis *serovars targeted in the L1-, L2- and L3-Real-time PCR.** (b) Alignments of the respective *omp*-1-sequences of the serovars A to L grouped in the L1-, L2- or L3-cluster. The accession numbers of the serovars are indicated. Identical nucleotides are boxed, the binding sites of primers and probes are marked above.

We added a control plasmid, IK_C, to each PCR reaction to monitor a possible inhibition of the PCR reaction. As both the potential target *C. trachomatis*-DNA of a sample and the added internal control DNA compete for the CT-primers, an absence of detection of the internal control by the Tex Red-labelled probe was tolerated in samples with a positive CT-detection, but indicative of PCR inhibition in specimens negative for *Chlamydia trachomatis*. The inclusion of the internal control did not interfere with the sensitivity of the CT/LGV PCR and led to a maximal increase of Ct-values of 2 cycles (Table 2). In all reactions with less than 10^5 ^copies of CT-amplicon the internal inhibition control plasmid was detected.

**Table 2:** Ct-values of CT- and (+/-) LGV- positive specimens tested in real-time PCRs in dependence on multiplexing

Sample	Specimen	CT (+/-IK_C)^a^	LGV (+/-IK_C)^a^	IK_C	L1 (+/-L2)	L2 (+/- L1)	L3 (+/-GAPDH)
L1	DNA	26/26	28/27	38	28/28	42/-	-/-
L2	DNA	27/26	27/27	42	-/-	28/30	-/-
L3 (dilut.)	DNA	34/34	33/35	32	-/-	-/-	34/35
04/256	Ocular	34/36	-/-	37	42/-	-/-	42/-
04/270	Urethral	-/-	-/-	36	40/-	-/-	-/39
04/1180	Rectal	26/26	-/-	36	-/-	-/-	-/-
06/3013	Rectal	35/36	-/-	36	-/-	-/-	-/39
04/234	Rectal	23/23	25/24	-	-/-	26/28	-/-
04/227	Rectal	32/31	34/33	34	-/-	35/36	-/-
04/261	Rectal	31/30	34/33	-	-/-	38/37	-/-
04/985	Rectal	31/30	34/32	34	-/-	34/32	40/-
05/697	Rectal	29/29	31/31	36	-/-	33/33	36/35
06/567	Urethral	nt/34	nt/34	36	-/-	36/37	-/-
06/1507	Rectal	28/28	31/31	35	-/-	31/34	-/39

^a ^CT/LGV-real-time PCR with (+) and without (-) addition of the internal control plasmid IK_C

**Table 3:** Ct-values of C. trachomatis serovars in CT/LGV-, L1/L2- and L3/GAPDH- real-time PCR

Species/Serovars	CT	LGV	L1	L2	L3	GAPDH
C. pneumoniae	-	-	-	-	-	36
C. psittaci	-	-	-	-	-	34
C. pecorum	-	-	-	-	-	36
C. muridarum	-	-	-	-	-	-

C. trachomatis						
B	31	37	-	-	-	-
D	25	-	-	-	-	31
D-	23	-	-	-	-	30
Da	24	-	-	-	-	28
E	31	-	-	-	-	26
F	36	-	-	-	-	24
G	30	-	-	-	-	25
Ga	21	-	-	-	-	29
H	29	-	-	-	-	31
Ia	24	-	-	-	35	-
I	21	-	-	-	37	26
J	25	-	-	-	-	30
K	24	-	-	-	-	34
L1	26	28	28	42	-	34
L2	27	27	-	28	-	-
L3	18	19	-	-	22	-
K-	-	-	-	-	-	-

### Differentiation of the LGV serotypes L1, L2 and L3

In order to develop a real-time PCR assay to detect and discriminate between L1-, L2- and L3-serotypes, a multiple sequence alignment of the *omp-1 *genes revealed a less conserved region which showed a 3'-overlap with the formerly described variable segment VS2 of *omp-1 *[7]. As shown in a phylogenetic tree of this gene region (Figure 3(a)), the serotypes of *C. trachomatis *cluster in three main branches:

• the L1-cluster containing the serotypes L1, D, Da, E, F and G;

• the L2-cluster containing the L2-serotypes (L2, L2a and L2b) and B/Ba; and

• the L3-cluster comprising the serotypes A, C, H, I/Ia, J/Ja, K and L3.

The designed L1-, L2- and L3-TaqMan PCR assays differed slightly in their relative positions within that gene region (Figure 3(b)). We tested the sensitivity on amplicons of the respective PCR, which had been cloned into a plasmid. The monoplex L1-, L2- and L3-PCR assays were similar in terms of efficiency (92.6% (L1), 95.3% (L2) and 93.5% (L3)), r^2 ^values (0,987, 0,998 and 0,995 resp.), and linear ranges between 10^9 ^and 10^2 ^copies per reaction. The detection limits corresponded with 50 genome copies per reaction to those of the CT/LGV duplex PCR. Analysing DNA of the serovars L1, L2 and L3 the Ct-values remained nearly un-affected when duplexing the HEX-labelled L1- with the FAM -labelled L2-reaction; and the TET-labelled L3- with the FAM-labelled GAPDH-PCR (Table 2). It should be noted that the fluorescence of the TET-labelled probe was detectable in the HEX-channel and in addition of nearly the same intensity in the FAM-channel. The amplification of human GAPDH provided a control of the quality of the specimen. When duplexed with the L3-PCR the value of the GAPDH-reaction was increased slightly in the presence of a positive L3-reaction, whereas the L3-value remained unaffected. To circumvent this problem in a multiplex assay, another fluorophore (e.g. TexasRed) should be used.

### Specificity of the real-time PCR assays

The analytical specificity of the multiplex CT/LGV-, L1/L2- and L3/GAPDH- real-time PCR assays was investigated by testing the *C. trachomatis *serovars and other bacteria that normally reside in the human perianal and urogenital tract (see Materials and Methods). There was no cross-reaction with DNA of non-chlamydial microorganisms (data not shown) or with chlamydial species other than *C. trachomatis *(Table 3). Analyses of the different *C. trachomatis *serovars revealed a high specificity of the newly developed real-time PCRs. However the serovars B, I-/Ia and L1 cross-reacted slightly, in that serovar B reacted in the LGV-reaction, I-/Ia in L3-PCR and L1 in L2-PCR. As the Ct-values of the specific and non-specific reactions differed by a factor of six or more, cross-reactions can be readily identified. Thus, the basic prerequisite for LGV-serotyping is a positive LGV-PCR with a Ct-value comparable to that of the CT-reaction. Weak cross-reactivity of L1-serovar in L2-PCR was a disadvantage when duplexing the typing assays. A highly positive L1-reaction and weak L2-PCR could either be based on a double infection with a high load of L1-serovar and a low load of L2-serovar or the result of the cross reacting L1-DNA in the L2-PCR. To avoid misinterpretation in this case the specimen must be analysed either in monoplex L1- and L2-PCR assays or subsequently in monoplex L2-real time PCR.

### Retrospective analysis of C. trachomatis -positive specimens

From 2004 to 2006 2381 ocular, urogenital and rectal specimens were tested by monoplex CT-real time PCR for *C. trachomatis *in the diagnostic laboratory. More than 90% of the tests were conducted on specimens from patients with clinical signs of infection. Of the 67 CT-positive specimens 52 samples were still available for retrospective analysis of the LGV status. We analysed these specimens in the CT/LGV duplex real-time PCR assay and found 50 positive for *C. trachomatis*. They consisted of ten conjunctival swabs, two poch of Douglas aspirations, eleven cervix swabs, 2 tubal swabs, fourteen urethral swabs and eleven rectal swabs. Two specimens, one urine and one cervix swab were negative. This discrepancy may be either due to a false-positive IFT or more probably to degradation of the DNA as an in-house nested PCR targeting 800 bp of the *omp*1 gene was also negative.

As summarised in Table 2, seven of the 50 *C. trachomatis *positives were also positive in LGV detection, all from men (six rectal and one urethral swab). With comparable Ct-values in both reactions a double infection with LGV- and non-LGV-serovars could be excluded. The rectal swabs were taken from HIV-positive patients, four of whom had ulcerating proctitis. The urethral specimen came from a HIV-negative patient with an urethral ulcer. LGV typing revealed that six were of L2-type. One rectal specimen revealed a double-infection with L2- and L3-serovars (Table 2). Sequencing of the L3-amplicon of the double positive specimen confirmed the presence of L3-DNA. As the sequence of the L2-amplicon is identical in L2-, L2a- and L2b-serovars and therefore unqualified for further sub-typing the L2-positives were subjected to a conventional PCR amplification of an extended region of *omp-1 *including point mutations between the subtypes. Sequence analysis of the PCR-products revealed that they were all of L2b-subtype.

## Discussion

The LGV-serotypes of *C. trachomatis *are emerging pathogens in the industrial world for men who have sex with men, often in association with HIV co-infection. The LGV-serovars cause acute illness, may persist for extended periods and, if untreated, may facilitate the spread of HIV. LGV clinical care, surveillance and research are severely hindered by the lack of widely available, rapid, standardized tests for the diagnosis of LGV.

Recently, a number of PCR-based test systems were published facilitating the identification of the LGV-associated L-serovars. Some of these dealt with the detection und typing of nearly all serovars of *C. trachomatis *in a given sample. For this purpose the groups of Molano [11] and Xiong [12] developed PCR-based reverse line blot (RLB) hybridisation tests and Quint and coworker used a PCR-based microplate reverse hybridisation assay (RHA) [33]. These methods were highly specific and suitable for the detection of mixed *C. trachomatis *serovars in a specimen. However, these methods require handling of the PCR products for subsequent analyses which is a possible source of contamination and therefore to be used with caution. This is also true of a two-step nested real-time PCR developed by the group of Jalal in 2007 which targeted the *omp*-1 gene and used eleven probes for genotyping the serovars D-K and L1, L2 and L3 in the second, nested step [34]. Modifying the conditions to a single-tube nested amplification would create a rapid, safe and sensitive method for genotyping *C. trachomatis*. Considering the disadvantages of post-PCR amplicon handling as well as the costs of these assays, these methods seem at the moment to be more suitable for epidemiological studies rather than the routine diagnostic laboratory.

The discrimination of LGV-serotypes from the other serovars of *C. trachomatis *is of major clinical importance as different antibiotic treatment regimens are required [35]. Detection is often based on amplification of the well known *omp-1 *gene, which encodes the major outer membrane protein, whereas other nucleic acid-based applications have used the *pmp-H *gene, in which the unique deletion in the LGV-serovars facilitates the design of an LGV-specific PCR, or the species-specific cryptic plasmid of *C. trachomatis*.

The group of Limberger evaluated a duplex real-time PCR based on the amplification of the cryptic plasmid (*C. trachomatis*-specific) and the *omp-1 *gene (serovar L2) [26]. Targeting the cryptic plasmid increases the sensitivity 10-fold, as there may be up to 10 plasmid copies per cell [16]. However, deletions found in the cryptic plasmid of some isolates were shown to affect nucleic acid amplification tests used in Sweden [36]. These findings suggest that detection of more than one gene of *C. trachomatis *may be necessary to avoid false-negative results due to mutation or recombination within the target sequences. Periodic checks of recently published mutations within the target gene regions will minimise the risk of decreased sensitivity.

In 2006 Goldenberger *et al*. published the evaluation of two real-time and one conventional PCR which targeted *omp-1*, the first two did not discriminate between L2 and L2b, whereas the latter was L2b-specific [25]. In our study we developed and evaluated a duplex real-time PCR for the simultaneous detection of *C. trachomatis*, targeting the *omp-1 *gene, and identification of the LGV-serotypes by targeting the deletion-spanning *pmp-H*-gene region. Completed by an inhibition control plasmid this assay fulfils the requirements of a specific routine diagnostic method. Although not as sensitive as the nested real time PCR assays described above [34] the sensitivity of this assay corresponds to that of Halse and coworkers [26] and was shown to exceed a commercial immuno-fluorescence test which is still considered the gold standard.

In a retrospective analysis of 50 ocular, rectal and urogenital specimens which were known to be positive for *C. trachomatis *we found seven positive for an LGV-serotype. Most of the specimens were obtained from HIV positive MSM. With comparable Ct-values in both reactions, CT and LGV, a double-infection with non-LGV-serovars was unlikely. With the newly developed *omp-1*-based real-time PCR assays we were able to discriminate between the LGV-serovars L1, L2 and L3 and to subtype the seven LGV-positives: six L2-positive and one L2/L3 positive specimen. Sequence analyses revealed that the seven L2-serovars detected in our study were all L2b-subtype, which is the most common LGV-serotype found in European MSM [23].

This is, to our knowledge, the first description of a differentiation of the three main LGV-serovars by the use of one-step real-time PCR assays which additionally enabled the discovery of a L2/L3 double-infection in a rectal specimen of a European MSM.

Genotyping analyses of *C. trachomatis *have revealed that recombination has also led to mosaic-variants of two serotypes [5-7]. Three L1/L2-variants have been described which carry mosaic *omp-1*-genes in which recombination in the variable region VS2 occurred [6] a mere 2 bp downstream of the L1-amplicon in our PCR (see Figure 3). Such mosaic recombinants would be indistinguishable from normal L1-serotypes in the L1-real-time PCR. A L1-serovar has already been detected in an MSM with proctitis [28] and detection of an L1/L2-mosaic in such patients will, in the future, rely on the sequencing of the downstream region. Nevertheless, although Gomes and co-worker [37] have found that *C. trachomatis *is prone to widespread recombination an altered pathogenicity has not been correlated to any specific recombination event. Regarding the higher virulence of LGV-serotypes in contrast to the other urogenital serovars, it is interesting that in a newly developed multi-locus sequence typing method for *C. trachomatis *ten specimens from MSM were identified as *omp*-1 genotype G [38].

The L2/L3 double infection which was detected in a European MSM by our study may be the pre-requisite for the creation of a new mosaic-variant. To take this into account, future epidemiological studies should be performed by methods that enable the detection of double-infections and include screening for new recombinants.

To validate the real time PCR assays presented here we will continue to sequence all new LGV-positives in future work.

## Conclusion

The two sets of real-time PCRs presented in this study enable the rapid identification and differentiation of the LGV-serovars L1, L2 and L3. The CT/LGV multiplex PCR is a useful tool for the routine diagnostic of specimens from MSM. For epidemiological studies this test can be upgraded by the assay to differentiate the LGV-serotypes L1, L2 and L3.

## Availability and requirements

National Center for Biotechnology Information: 

## Competing interests

The authors declare that they have no competing interests.

## Authors' contributions

AS carried out most of the PCR assays. BH designed the study and drafted the manuscript. Both authors have read and approved of the final manuscript.

## Pre-publication history

The pre-publication history for this paper can be accessed here:


